# The Burden of Statin Therapy based on ACC/AHA and NCEP ATP-III Guidelines: An Iranian Survey of Non-Communicable Diseases Risk Factors

**DOI:** 10.1038/s41598-018-23364-9

**Published:** 2018-03-21

**Authors:** Samaneh Asgari, Hengameh Abdi, Alireza Mahdavi Hezaveh, Alireza Moghisi, Koorosh Etemad, Hassan Riahi Beni, Davood Khalili

**Affiliations:** 1 Prevention of Metabolic Disorders Research Center, Research Institute for Endocrine Sciences, Shahid Beheshti University of Medical Sciences, Tehran, Iran; 2 Endocrine Research Center, Research Institute for Endocrine Sciences, Shahid Beheshti University of Medical Sciences, Tehran, Iran; 3 Center for Non-communicable Disease Control, Ministry of Health and Medical Education, Tehran, Iran; 4 Environmental and Occupational Hazards Control Research Center, Shahid Beheshti University of Medical Sciences, Tehran, Iran; 5 Cardiovascular Department, Rasoul e Akram General Hospital, Iran University of Medical Sciences, Tehran, Iran

## Abstract

To compare the burden of statin therapy according to the Third Adult Treatment Panel (ATP-III) and the American College of Cardiology/American Heart Association (ACC/AHA) guidelines the Survey of Risk Factors of Non-Communicable Disease (SuRFNCD)−2011of Iran was used. A survey analysis associated with sex and age categorization was run. Of total 3496 persons (1322 men) aged 40–70 years, based on the ACC/AHA guidelines, about 46.5% were eligible to receive moderate- to high-intensity statin therapy. Based on the ATP-III guidelines, 17.0% were considered as needing statin drugs. Among adults aged <60 years, the proportion of those who were eligible for statin therapy was higher (38.3%) according to the ACC/AHA guidelines compared to the ATP-III guidelines (15.2%), a difference more prominent in adults aged ≥60 years (85.2% versus 25.0%). Agreement between the two guidelines was low (kappa: 0.32). Compared to the ATP-III guidelines, the ACC/AHA guidelines increase the number of adults eligible for statin therapy in an Iraninan population from 2.5 million to 7.0 million people according to the 2011 census, specifically in those aged ≥ 60 years, a finding in agreement with those of studies from different countries.

## Introduction

From decades ago, hypercholesterolemia has been recognized as an important and modifiable risk factor for cardiovascular diseases (CVDs)^1,2^. Several qualified meta-analyses have documented that cholesterol reduction with statins decreases CV morbidity and mortality in people with different baseline CV risk^3–5^; therefore, most guidelines recommend lipid-lowering therapy to prevent CVD.

The Adult Treatment Panel III (ATP-III) guidelines from the National Cholesterol Education Program (NCEP) for management of hypercholesterolemia were issued in 2001 and modified in 2004. In this report, Framingham 10-year risk for hard coronary heart disease (CHD) events was used for categorizing persons into 3 categories of high risk (>20%), moderate risk (10–20%) and low risk (<10%). Regarding drug therapy, patients with prevalent CHD or CHD risk equivalents are eligible for low density lipoprotein (LDL-C)-lowering drugs if they have LDL-C ≥100 mg/dl. Other individuals should receive statin therapy based on their risk category and LDL-C levels^6,7^.

In 2013, the American College of Cardiology (ACC) and the American Heart Association (AHA) released new guidelines on the assessment of CV risk and management of hypercholesterolemia^8,9^, which have been criticized from differ ent perspectives^10–13^. Main comments on these guidelines were regarding new perspectives on LDL–C treatment goals, use of a new CV risk assessment tool and threshold of 5% for moderate-intensity statin therapy. The latter change can increase the number of eligible individuals for statin therapy, imposing a heavy burden on health budgets. Based on the ACC/AHA guidelines, over one billion healthy (without CVD, diabetes or high LDL-C) people worldwide are estimated to receive statin and the global cumulative sales of statins by 2020 will be about a trillion dollars^10,14–16^.

Considering these potential implications, differences between the two cholesterol guidelines encouraged us to compare the burden of statin therapy according to these guidelines in the national survey of risk factors of non-communicable diseases (SuRFNCD) in Iran.

## Results

**Figure 1:**
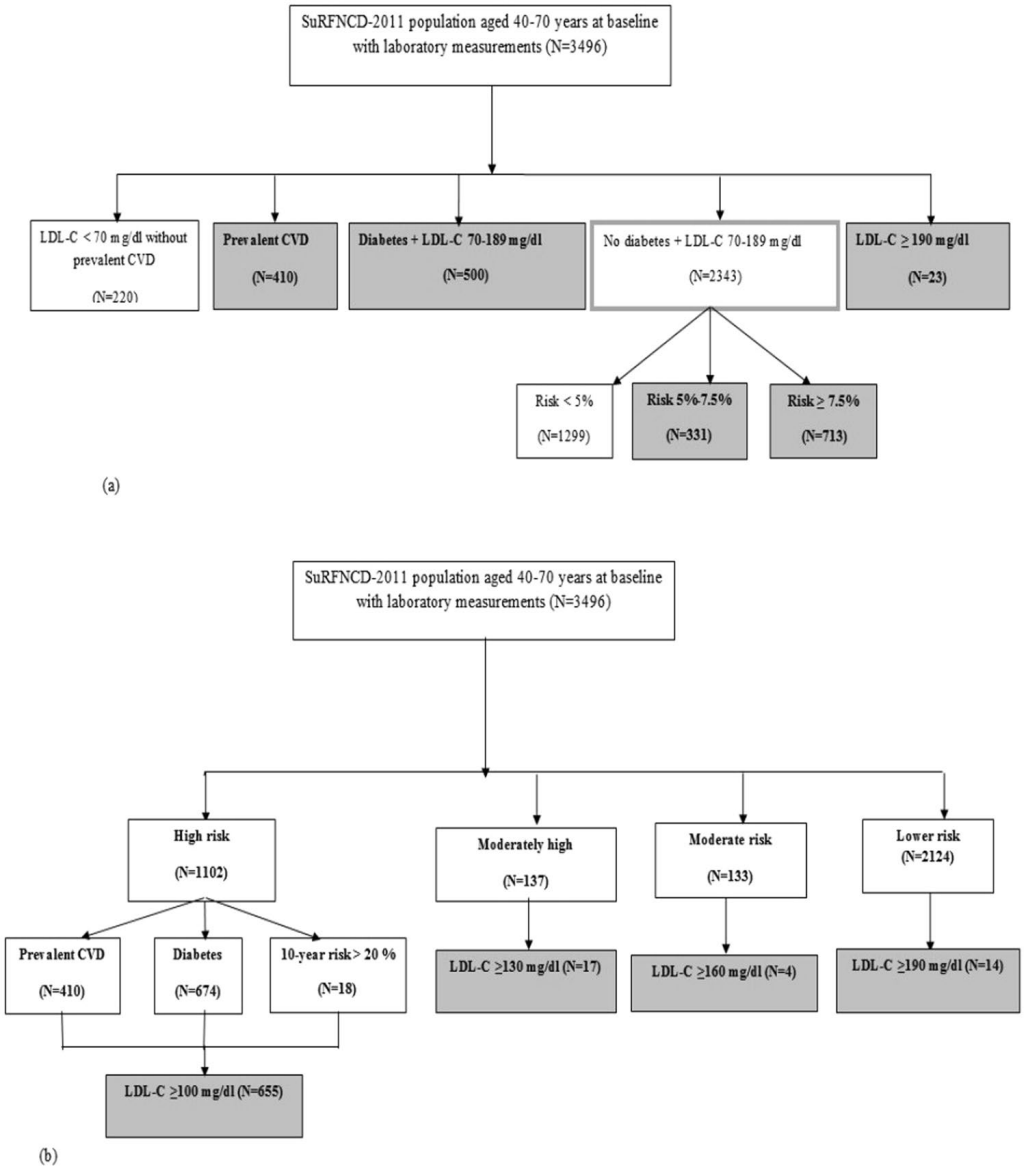
(**a**) Study population considering individuals who are eligible for lipid lowering therapy (1977) based on the American College of Cardiology/American Heart Association (ACC/AHA) cholesterol guidelines. Subcategories that need treatment are highlighted. CVD, cardiovascular disease; LDL-C, low density lipoprotein cholesterol. (**b**) Study population considering individuals who are eligible for lipid lowering therapy (690) based on Framingham risk score for the Third Adult Treatment Panel (ATP-III) guidelines. Subcategories that need treatment are highlighted. CVD, cardiovascular disease; LDL-C, low density lipoprotein cholesterol.

Of 11867 individuals, complete data of 3496 individuals, aged 40–70 years was available; of these, 2174 (50.2%) were female. Mean (SE) age of the study population was 51.42 (0.06) years. Based on the ACC/AHA guidelines, 1977 (46.5%) of the study population [1019 (39.3%) female] were eligible to receive moderate- to high-intensity statin therapy [Fig. 1(a)]. Based on the ATP III guidelines, of the total study population aged 40–75 years, 690 (17.0%) subjects comprising of 417 females and 273 males, were believed to need statin drugs [Fig. 1(b)]. Table 1 demonstrates characteristics of the total population and those eligible for statin therapy according to each set of the two guidelines, separately. Characteristics of individuals eligible for statin therapy considering the ATPIII guidelines were compared with those of individuals eligible considering the ACC/AHA guidelines but not based on the ATP III guidelines; as displayed in Table 1, the latter individuals were older persons with better cardiovascular risk profile.

**Table 1:** Cardiovascular risk profile of study population: Iran SuRFNCD-2011data.

	Total (N = 3496)	Adults eligible for statin therapy^a^			
		ACC/AHA guidelines (N = 1977)	ATP III guidelines (N = 690)	ACC/AHA but not ATP III guidelines (N = 1287)	p-value^b^
Female gender, n (%)	2174(50.25)	1019(39.34)	417(50.04)	602(33.2)	<0.001
Age, year	51.42(0.06)	51.97 (0.1)	51.75(0.17)	52.1(0.16)	0.13
Age category, n (%)					<0.001
-40–59 yr	2338(82.52)	1006(68.01)	402(74.2)	604(64.5)	
-≥ 60 yr	1158(17.47)	971(31.99)	288(25.8)	683(60.2)	
BMI, kg/m^2^	27.81(0.26)	28.5(0.31)	28.5(0.32)	28.5(0.5)	0.65
SBP, mm Hg	131.64(0.88)	137.62(1.24)	139.8(1.7)	136.5(1.4)	0.49
DBP, mm Hg	82.25(0.47)	85.22(0.65)	86.2(0.95)	84.7(0.7)	0.34
Total cholesterol, mg/dl	189.67(1.67)	202.0(2.3)	221.6(2.96)	184.5(2.6)	<0.001
HDL-C, mg/dl	44.78(0.48)	43.0(0.57)	43.2(0.74)	42.6(0.72)	0.74
LDL-C, mg/dl	106.2(1.09)	113.0(1.3)	127.5(1.6)	100.0(1.2)	<0.001
Triglyceride, mg/dl	161.56(3.35)	193.2(5.4)	209.3(9.0)	182.0(8.2)	0.15
FPG, mg/dl	105.6(1.24)	127.6(3.0)	142.5(4.7)	115.7(4.1)	0.02
Diabetes, n (%)	674(15.9)	635(32.16)	401(55.33)	234(18.9)	<0.001
Hypertension, n (%)	1774(43.6)	1263(59.13)	473(66.2)	790(55.1)	0.004
Prevalent CVD, n (%)	410(9.8)	410(21.05)	222(32.2)	188(14.7)	<0.001
Medication use, n (%)					
-Hypertension drug	973(20.8)	746(31.5)	287(36.17)	459(28.8)	0.45
-Diabetes drug	449(10.0)	422(30.31)	244(33.06)	178(13.0)	<0.001

SuRFNCD, survey of risk factors of non-communicable diseases; ACC/AHA, American College of Cardiology/American Heart Association; ATPIII, Third Adult Treatment Panel; BMI, body mass index; SBP, systolic blood pressure; DBP, diastolic blood pressure; HDL-C, high density lipoprotein cholesterol; LDL-C, low density lipoprotein cholesterol; FPG, fasting plasma glucose; CVD, cardiovascular disease.

Mean (SE) are shown for continuous variables.

^a^Column percentages based on survey analysis are reported.

^b^The reported p-value is based on the differences between individuals eligible for statin therapy considering the ATPIII guidelines and those eligible considering the ACC/AHA guidelines but not based on the ATP III guidelines.

**Figure 2:**
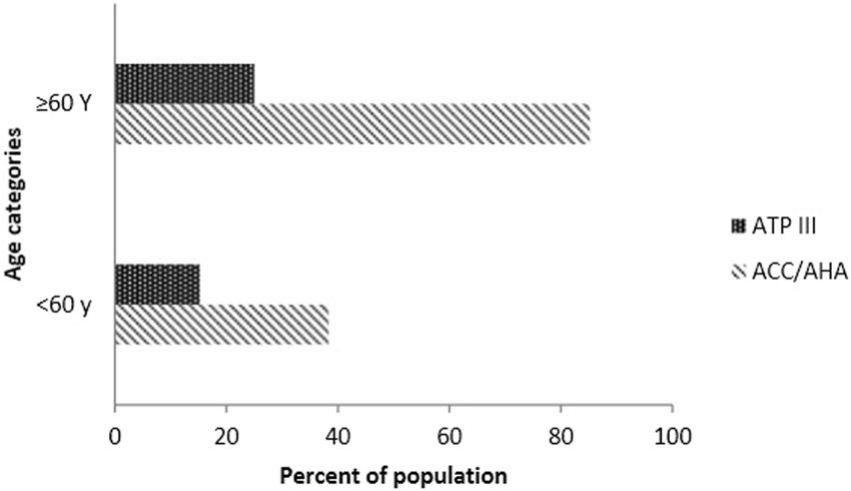
Percent of Iranian adults who are eligible for statin therapy based on the Third Adult Treatment Panel (ATP-III) and the American College of Cardiology/American Heart Association (ACC/AHA) cholesterol guidelines according to age groups.

As shown in Fig. 2, among adults aged <60 years, the proportion of those who were eligible for statin therapy was higher (38.3%) based on the ACC/AHA guidelines vs the ATP III guidelines (15.2%). This difference was more prominent in adults aged ≥60 years (85.2% according to the ACC/AHA guidelines versus 25.0% according to the ATP III guidelines).

All 690 participants who were eligible for receiving statin therapy based on the ATP-III guidelines were also eligible to be treated according to the ACC/AHA guidelines, whereas, 1287 (63.64%) participants, including 685 men (50%) and 602 women (50%), were candidates for statin therapy only with respect to the ACC/AHA guidelines. The results showed a low level of agreement between two guidelines (Kappa agreement, 0.32) (Table 2).

**Table 2:** Distribution and Kappa agreement of lipid lowering therapy based on the ACC/AHA and ATP-III guidelines.

		Lipid lowering therapy based on ATP-III guidelines	No	Kapp agreement	p-value
		Yes			
Lipid lowering therapy based on ACC/AHA guidelines	**Yes**	690(36.36)	1287(63.64)	**31.8%**	**<0.001**
	**No**	0	1519(100.0)		

ACC/AHA, American College of Cardiology/American Heart Association; ATPIII, Third Adult Treatment Panel.

## Discussion

In this national study, the eligibility for statin therapy with respect to the ACC/AHA guidelines on the management of blood cholesterol with the aim of primary prevention of CVD was compared with the ATP-III guidelines, based on the survey analysis. Compared to the ATP-III guidelines, the recent guidelines increase the number of eligible participants for statin therapy (46.5% vs 17.0%). Considering age group analyses, this difference was more significant in participants aged ≥60 years (25.0% vs. 85.2%).

With respect to the Tehran Lipid and Glucose Study (TLGS), in an urban population of Iran, the burden of statin therapy according to the ACC/AHA guidelines was estimated to be about 8 million adults based on the 2011 census^17^ i.e. about 57% of healthy population, aged 40–75 years. Several studies have reported that the ACC/AHA guidelines increase the proportion of eligible individuals for statin therapy. Pencina *et al*.^18^ showed that nearly 48% of participants, aged 60–75 years would be eligible for statin therapy based on the ATP-III guidelines, whereas this amount increases to 77% based on the ACC/AHA guidelines; albeit in individuals aged 40–59 years, percentages of eligible candidates were similar. Recently, Cho *et al*.^19^ reported a higher proportion of eligible candidates for statin therapy according to the ACC/AHA guidelines compared to the ATP-III guidelines in a Korean population (55.0% vs 20.5%); this increase was more evident in people aged >60 years (84.5% vs 26.1%). Overall, it should be taken into account that the risk assessment tools of the two guidelines differ from each other and the ACC/AHA uses a pooled cohort equation to estimate both CHD and stroke events which their prevalence increases in the older population.

In the present study, individuals eligible for statin therapy according to the ACC/AHA guidelines but not based on the ATP III guidelines were older persons with better cardiovascular risk profile. With regard to more significant difference between the two guidelines in the elderly, it should be kept in mind that according to more benefit of cholesterol lowering medications in the elderly, this age group population with high rate of CVD events and mortality, should not be deprived of statin therapy; however, many considerations including comorbidities, life expectancy, drug interactions due to polypharmacy should be taken into account^20,21^.

We also attempted to estimate the level of agreement between these two guidelines on eligible participants for statin therapy and found to be low (Kappa = 0.32, p-value < 0.001). In the Jung, *et al*. study in a Korean population with subclinical coronary atherosclerosis^22^, the level of agreement was 45% which was markedly higher than our study, which may be due to different study populations regarding their baseline risk.

Although statins decrease the risk of cardiovascular events, concerns about side effects, especially muscle-related, particularly in situations where the number of eligible candidates for statin therapy increases according to the ACC/AHA guidelines, may dissuade physicians from prescribing these beneficial medications^23,24^. However, with respect to new-onset diabetes (DM) following statin use, data from clinical trials^25,26^ also observational studies^27^ show only modestly increased risk of DM, especially in individuals with risk factors of DM^28^. Regarding other adverse effects, a systematic review of randomized statin trials showed that statin therapy slightly increases the risk of transaminase elevations, although not myalgia, creatine kinase elevations, rhabdomyolysis, or withdrawal of therapy compared with placebo^29^. Available data addressing the effects of lipid lowering with statins on the neurocognitive function is conflicting^30^. Taken together, considering the safety aspects of statins plus the significant reduction in the risk of CVD events, benefits of statin therapy may outweigh the high proportion of people^31,32^.

Our study has some limitations. First, we could not obtain participants’ information on lipid lowering therapies which can cause overestimations in our findings. In the Tehran Lipid and Glucose Study (TLGS), only 6% of the 40–75 year-old urban Iranians used lipid lowering drugs^32^. Second, we did not have detailed data on history of CHD, cerebrovascular events or peripheral vascular disease and used only prevalent CVD data that might result in the underestimation of CVD. Last but not least is the lack of information on family history of premature CVD which is one of risk factors included in the ATP-III risk assessment algorithm. However, the major strength of this study lies in its large sample size from a national Iranian population (SuRFNCD-2011).

In conclusion, compared to the ATP-III guidelines, use of the ACC/AHA guidelines increase the number of adults eligible for statin therapy in the Iranian population from 2.5 million to 7.0 million people according to the 2011 census, specifically in those aged ≥60 years. These findings are in agreement with those of other studies from different countries.

## Materials and Methods

### SuRFNCD-2011

We conducted a secondary analysis on data of SuRFNCD-2011. Briefly, SuRFNCD-2011 is a national survey for non-communicable disease risk factors using a random multistage cluster sampling method in non-hospitalized and non-institutionalized Iranian individuals, aged 6–70 years^33^. Between May 22^nd^ and June 20^th^ 2011, 11,867 individuals, including 4955 men were surveyed using a four stage sampling system. Each address was obtained and the individual was contacted and registered. Non-response was reported for an individual sampling level if he/she refused to participate after three attempts. A consent form with respect to the Declaration of Helsinki was read out by the interviewer at the beginning of each interview and acceptance or refusal to participate was officially documented. All participants provided verbal informed consent. The CDC Board of Ethics also approved the study protocol^33^. All methods of the current study were performed in accordance with the relevant guidelines and regulations.

### Medical history, clinical examination and laboratory measurements

All experiments were performed in accordance with relevant guidelines and regulations. Systolic blood pressure (SBP) and diastolic BP (DBP) were measured three times, with intervals of 5 minutes, in a seating position from the right arm using a standard Omron M7 sphygmomanometer (HEM-780-E) and the mean value was considered as the subject’s SBP and DBP. All measurements of lipid concentrations were assessed in venous samples drawn after 12–14 h overnight fasting. According to the standard protocol, a 10 ml sample of venous blood, collected in 4 tubes, was drawn from each individual by trained laboratory staff and was sent to collaborating centers. Samples were immediately centrifuged (1500 rpm for 10 min) at each designated laboratory, and transferred under cold chain conditions to the Central Reference Laboratory of Ministry of Health of Iran (Tehran, Iran). Serum lipids including total cholesterol, triglycerides, LDL-C and high density lipoprotein (HDL-C) were determined by enzymatic methods (Parsazmun, Karaj, Iran). Enzymatic calorimetric methods with a glucose oxidize test was used for measuring fasting plasma glucose (FPG). Inter- and intra-assay coefficients of variation (CV) at baseline and follow-up phases were 2.1% and 2.6%, respectively^33^.

### Definition of terms

Type 2 diabetes (DM) was defined if the participant responded positively to any of following questions: “Are you currently taking oral agent or insulin for DM, prescribed by a doctor or other health worker?” and “Have you ever been told by a doctor or other health worker that you have diabetes?” or if his/her FPG was ≥126 mg/dl. Hypertension was defined as SBP ≥ 140 mmHg or DBP ≥ 90 mmHg or taking any anti-hypertensive medications.

A current smoker was defined as a person who was smoking daily any amount of any kind of tobacco at the time of the interview. Daily smokers of pipe and pipe/chopogh users were also defined as current smokers.

Prevalent CVD was defined as responding positive to the following question: “Have you ever been told by a doctor or other health worker that you have a heart problem?”

### ATP-III risk assessment

The NCEP ATP-III guidelines, professional guidelines on detection, evaluation and treatment of hypercholesterolemia were published in 2001 and modified in 2004^6,7^. In this report, for determination of LDL-C goals and cut points for therapy, four subgroups of individuals aged >20 years have been defined: (1) High risk: subjects with prevalent CHD or CHD risk equivalent which includes non-coronary forms of atherosclerotic disease, DM or having ≥2 risk factors with 10-year risk for hard CVD >20%, (2) Moderately high risk: subjects having ≥2 risk factors with 10-year risk for hard CVD 10% to 20%, (3) Moderate risk: having ≥2 risk factors with 10-year risk for hard CVD <10%, and (4) Low risk: 0–1 risk factor. Ten-year CV risk scoring was based on the Framingham risk scoring method; risk factors included in this algorithm are age, gender, cigarette smoking, family history of premature CHD, hypertension (SBP ≥140 mmHg or DBP ≥90 mmHg or antihypertensive treatment), low-HDL-C (<40 mg/dl) and total cholesterol.

According to the ATP-III categorization, recommended LDL-C levels for drug therapy are ≥ 100 mg/dl, ≥ 130 mg/dl and ≥160 mg/dl in the high-, moderately high- and the moderate risk groups, respectively. Lower risk persons will receive treatment if LDL-C level is ≥ 190 mg/dl.

### ACC/AHA risk assessment

The 2013 ACC/AHA guidelines^34^ used the Pooled Risk Equations for non-Hispanic white and black men and women to assess CV risk. The 10-year risk of hard CVD events is calculated based on age, gender, race, SBP, DBP, treatment for hypertension, total cholesterol, HDL-C, DM and current smoking status. Individuals, aged 40–75 years are categorized as four high risk subgroups that benefit from moderate- to high-intensity statin therapy as follows: (1) High-intensity statin for individuals with clinical CVD, (2) High-intensity statin for subjects with LDL-C ≥ 190 mg/dl, (3) High-intensity statin for diabetic patients with LDL-C 70–189 mg/dl and a 10-year CVD risk of at least 7.5% and moderate-intensity statin for those with CVD risk <7.5%, and (4) Moderate- to high-intensity statin for non-diabetic individuals with LDL-C 70–189 mg/dl and 10-year hard CVD risk of at least 7.5% and moderate-intensity statin for those with CVD risk of 5–7.5%.

### Statistical analysis

Mean (SE) values for continuous and frequencies (%) for categorical variables of baseline characteristics were described. A survey analysis was done using Stata 14 for Windows to generalize the results to the adult Iranian population; data were weighted directly to the 2011 population of Iran aged 40–49 years, 50–59 years and ≥60 years, based on the 2011 national Iranian census, to match the age (10-year strata) and gender.

Kappa statistics were measured to determine the chance level of agreement between the ATP-III and ACC/AHA guidelines^35^. As suggested by Landis and Koch, kappa values of <0.40, 0.41 to 0.60, 0.61 to 0.8 and 0.81 to 1.0 would indicate poor-to-fair agreement, moderate agreement, significant agreement and excellent agreement, respectively^36^.
